# Relationship Between Pregnant Women's eHealth Literacy and Their Attitudes Toward Sexuality

**DOI:** 10.1002/brb3.70390

**Published:** 2025-02-28

**Authors:** Elif Çini, Hamide Aygör

**Affiliations:** ^1^ Konya City Hospital Konya Turkey; ^2^ Department of Obstetrics and Gynaecology Nursing, Nursing Faculty Necmettin Erbakan University Konya Turkey

**Keywords:** attitude, eHealth, pregnant women, sexuality

## Abstract

**Objectives:**

During pregnancy, which involves intense physiological and psychological changes, attitudes toward sexuality may change. eHealth literacy is highly important in this period, during which sexuality changes, for pregnant women to access information about sexuality, research such information, make effective decisions, and shape their attitudes toward sexuality. This study was planned to examine the relationship between the eHealth literacy levels of pregnant women and their attitudes toward sexuality.

**Methods:**

This is a cross‐sectional study. The study was carried out with the participation of 297 pregnant women who presented to the antenatal outpatient clinics of a university hospital. A personal information form, the eHealth Literacy Scale, and the Attitude Scale toward sexuality during pregnancy were utilized to collect data. In the analyses, the independent‐samples *t*‐test, Mann–Whitney *U* test, one‐way analysis of variance, Kruskal–Wallis *H* test, Pearson's correlation analysis, and multiple linear regression analysis methods were used.

**Results:**

The participants had a mean total eHealth Literacy Scale score of 25.28 ± 7.15, while their mean total Attitude Scale toward sexuality during pregnancy score was 118.87 ± 16.7. It was determined that as the eHealth literacy levels of the participants increased, their positive attitudes toward sexuality during pregnancy also increased. It was concluded that eHealth literacy is a significant positive predictor of the attitudes of pregnant women toward sexuality.

**Conclusions:**

To have the ability to protect and improve maternal and fetal health by making appropriate interventions, it is important for health professionals to know the information requirements and sources of pregnant women. It is recommended to determine the eHealth literacy levels of pregnant women and assess their sexuality‐related attitudes in their routine follow‐ups.

## Graphical abstract

A positive relationship was identified between attitudes toward sexuality during pregnancy and eHealth literacy. Attitudes toward sexuality during pregnancy are affected by eHealth literacy, age, and monthly income.

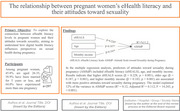

## 1 Introduction

As the internet is becoming more and more prevalent today, and access to the internet is more convenient, various forms of information can be reached on the internet (Al‐Dahshan et al. 2022; Artieta‐Pinedo et al. 2018; Hadımlı et al. 2018). The function of the internet as a facilitator of access to information in the field of health has given rise to the concept of eHealth literacy. Researchers define eHealth literacy as the “capacity to search for and find information related to health on electronic sources”, understand it, evaluate it, and use it to make decisions about one's health (Norman and Skinner 2006a). One of the groups of people who use the internet to access health‐related information and make decisions is pregnant women (Šoštarić and Jokić‐Begić 2020). The topics about which pregnant women prefer to reach information on the internet include pregnancy symptoms, fetal development, physical activity, pregnancy complications, childbirth, breastfeeding, and infant care. Sexuality is also among these topics (Dickerson 2006; Kamali et al. 2018). Supporting information s1

Healthy sexuality during pregnancy strengthens the compatibility of the couple and their emotional connection and plays a role in the continuation of their relationship. In periods like pregnancy where changes in sexuality are experienced, eHealth literacy is highly important for a healthy sex life. eHealth literacy levels are very important for pregnant women to access information about sexuality, research such information, make effective decisions, and shape their attitudes toward sexuality. For this reason, in prenatal follow‐ups, pregnant women should be evaluated in terms of their eHealth literacy levels, and their eHealth literacy should be improved (Abiş and Kantaş Yılmaz 2020; Nawabi et al. 2021; Meldgaard et al. 2022). In the literature, there are studies that have examined the eHealth literacy levels of pregnant women (Villadsen et al. 2020; Xu et al. 2024; Yumei et al. 2024) or their attitudes toward sexuality (Adegboyega 2019; Igbana et al. 2021; Fatima et al. 2022). However, no study in which the relationship between the eHealth literacy levels of pregnant women and their attitudes toward sexuality was investigated could be encountered. To fill this gap in the literature, this study was planned to determine the relationship between the eHealth literacy of pregnant women and their attitudes toward sexuality.

## 2 Materials and Methods

The study was designed as a cross‐sectional study.

### 2.1 Participants

The minimum required sample size for the study was calculated as 272 participants, based on a 95% confidence interval, a 90% testing power, and a 0.10 effect size (G*Power 3.1.9.2). The study was completed with 297 participants. The post hoc power analysis showed a power of 0.957.

Pregnant women who were carrying singleton and healthy fetuses, did not have any communication problems, and had access to electronic sources were included in the study. Those who had become pregnant as a result of treatment and those who had been instructed to avoid sexual intercourse by their doctors due to risky pregnancies were excluded.

### 2.2 Measures

The data were collected using a Personal Information Form, the eHealth Literacy Scale, and the Attitude Scale toward sexuality during pregnancy (AStSdP). The Personal Information Form consisted of questions about the sociodemographic (age, education status, partner's age, partner's education status, duration of marriage) and obstetric (number of pregnancies, number of living children) characteristics of the participants.

The eHealth Literacy Scale (eHEALS), which was developed by Norman and Skinner (2006b), was evaluated in terms of its reliability and validity in the Turkish language by Tamer Gencer (2017). This scale consists of two questions on internet usage that are not included in the scoring and eight items, which are rated based on a 5‐point Likert‐type scoring system. The scale does not have any inversely scored items or a cut‐off point. Possible scale scores vary between 8 and 40. Higher scores are considered to be indicative of greater eHealth literacy levels. The Cronbach's alpha coefficient of the scale was reported to be 0.915 in its Turkish validity and reliability study (Tamer Gencer 2017), while it was calculated as 0.951 in this study.

The AStSdP was created by Sezer and Şentürk Erenel to investigate the sexuality‐related attitudes of pregnant women or men whose partners are pregnant. AStSdP includes 34 items and three subscales: the “anxiety about sexual intercourse during pregnancy” subscale with 9 items, the “dysfunctional beliefs and values about sexuality during pregnancy” subscale with 10 items, and the “approving sexuality during pregnancy” subscale with 15 items. It is a 5‐point Likert‐type measurement instrument, with minimum and maximum scores of 34 and 170. While greater AStSdP scores are accepted to correspond to more positive sexuality‐related attitudes during pregnancy, lower scores indicate more negative attitudes. The cut‐off point of the scale is 111.5, and scores greater than 111.5 are interpreted as having positive attitudes regarding sexuality during pregnancy. The Cronbach's alpha coefficients of the subscales of the scale were reported to vary in the range of 0.81–0.86, while the coefficient for the total scale was 0.90 (Sezer and Şentürk Erenel 2021). In this study, this coefficient was determined to be in the range of 0.77–0.86 for the dimensions and equal to 0.88 for the overall instrument.

### 2.3 Data Collection

This study was carried out between June and August 2022 at the antenatal outpatient clinics of Necmettin Erbakan University Faculty of Medicine Hospital. Pregnant women who attended the antenatal outpatient clinics for routine follow‐ups and met the inclusion criteria were included in the sample. Women were included in the sample by nonprobability random sampling, and the data were collected face‐to‐face. It took about 15–20 min to collect data from each participant.

### 2.4 Ethical Considerations

Ethics committee approval was obtained from the Ethics Committee of Necmettin Erbakan University (Approval no. 221), and written permission was obtained from the hospital where the study was conducted. The purpose of the study was explained to the participants, and verbal informed consent was obtained from the participants.

### 2.5 Statistical Analysis

The statistical analyses were conducted using the Statistical Package for the Social Sciences (SPSS) 25.0. The descriptive statistics of the collected data consisted of frequency, percentage, mean, standard deviation, median, minimum, and maximum values. Mean AStSdP scores were compared between two groups of participants using the independent‐samples t‐test or the Mann–Whitney *U* test and among three or more groups using one‐way analysis of variance (ANOVA) (post hoc Bonferroni multiple comparisons) or the Kruskal–Wallis *H* test (post hoc Mann–Whitney *U* test for pairwise comparisons). The relationships between variables were analyzed using Pearson's correlation analysis method, and the predictive relationships between variables were analyzed using the multiple linear regression analysis (backward) method. The results of all analyses were evaluated within a 95% confidence interval.

## 3 Results

The distributions of the sociodemographic and obstetric characteristics of the participants are presented in Table 1. The participants had a mean total eHEALS score of 25.28 ± 7.15, while their mean total AStSdP score was 118.87 ± 16.73 (Table 2). Considering the questions on eHEALS that are not included in the scoring, for the question about the contribution of the internet while making decisions about one's health, 10.8% of the participants responded as “not useful at all,” 10.1% responded as “not useful,” 32.3% responded as “no idea,” 44.8% responded as “useful,” and 2% responded as “very useful.” For the other question that is not included in the scoring, regarding the importance of accessing health‐related sources on the internet, 5.7% of the participants responded as “not important at all,” 14.5% responded as “not important,” 22.9% responded as “no idea,” 49.5% responded as “important,” and 7.4% responded as “very important.”

**TABLE 1:** Sociodemographic and obstetric characteristics.

Variable	*n*	%
**Age groups (year)**		
17–25	124	41.8
26–35	136	45.8
≥ 36	37	12.4
**Educational status**		
Primary‐middle school	127	42.8
High school	110	37.0
University or higher	60	20.2
**Partner's age groups**		
17–25	65	21.9
26–35	170	57.2
≥ 36	62	20.9
**Partner's education status**		
Primary‐middle school	128	43.1
High school	96	32.3
University or higher	73	24.6
**Marriage duration**		
≤ 5 years	175	58.9
Ø 5 years	122	41.1
**Number of pregnancies**		
One	107	36.0
Two or more	190	64.0
**Number of living children**		
None	125	42.1
One	83	27.9
Two or more	89	30.0

**TABLE 2:** eHEALS and AStSdP scores.

Scales	Mean ± SD	Median	Min‐max
**eHEALS**	25.28 ± 7.15	27.00	8–40
**AStSdP (total)**	118.87 ± 16.73	119.00	40–167
**AStSdP dimensions**			
Anxiety about sexual intercourse	31.13 ± 7.03	32.00	9–45
Dysfunctional beliefs and values about sexuality	39.98 ± 5.86	40.00	15–50
Approving sexuality	47.77 ± 8.30	48.00	16–73

Abbreviations: AStSdP, attitude scale toward sexuality during pregnancy; eHEALS, eHealth Literacy Scale; Max, maximum scale score; Min, minimum scale score; SD, standard deviation.

There were weak, positive, and statistically significant relationships between the eHEALS scores of the participants and their total AStSdP scores, AStSdP “dysfunctional beliefs and values about sexuality during pregnancy” dimension scores, and AStSdP “approving sexuality during pregnancy” dimension scores (*p* < 0.01, Table 3). The eHEALS scores of the participants were very weakly related to their AStSdP “anxiety about sexual intercourse during pregnancy” dimension scores, but the relationship between these variables was not found to be significant (*p* > 0.05, Table 3).

**TABLE 3:** Relationships between eHEALS and AStSdP scores.

Scale	eHEALS	
	*r p*	
**AStSdP (total)**	0.233	**0.001**
**AStSdP dimensions**		
Anxiety about sexual intercourse	0.051	0.380
Dysfunctional beliefs and values about sexuality	0.168	**0.004**
Approving sexuality	0.308	**0.001**

Abbreviations: AStSdP, attitude scale toward sexuality during pregnancy; eHEALS, eHealth Literacy Scale; *r*, Pearson's correlation analysis.

P values less than 0.05 are considered statistically significant and are therefore bold.

The independent variables that were identified to have significant effects in the univariate analyses were entered into the multiple regression analysis. It was determined that the AStSdP scores of the participants were influenced by 5 independent variables (age, age of partners, marriage duration, monthly income, and eHEALS score). The results of the multiple linear regression analysis (backward method) on the relationships between these independent variables and the dependent variable are presented in Table 4. According to the correlation analysis results and collinearity statistics of the independent variables among each other, there was no autocorrelation problem in the data.

**TABLE 4:** Predictors of AStSdP (multiple regression analysis‐enter model).

Dependent variable	*β* ± SE	*t*	*p*	Collinearity Tolerance VIF	
Constant	± 6.185	13.097	**0.000**		
eHEALS	0.228 ± 0.130	4.105	**0.000**	0.963	1.038
Age of pregnant woman	0.187 ± 0.162	3.396	**0.001**	0.980	1.021
Monthly income	0.183 ± 1.849	3.301	**0.001**	0.972	1.029
*R*: 0.357 *R* ^2^:0.12 Adjusted *R* ^2^:0.112 *F*: 14.243 *p*: 0.000					

Abbreviations: AStSdP, attitude scale toward sexuality during pregnancy; eHEALS: eHealth Literacy Scale; SE, standard error.

P values less than 0.05 are considered statistically significant and are therefore bold.

Among the variables that were included in the regression model, two independent variables were removed from the model (first age of the spouse and then marriage duration) as they were not found to predict AStSdP scores significantly (*p* > 0.05, Table 4). For the variables that remained in the model as a result, the order of significance based on their *β* coefficients indicating their prediction of AStSdP scores was (from the most significant to the least significant) as follows: eHEALS score, age, and monthly income (*p* < 0.001). These three independent variables explained 12.7% of the total variance in the AStSdP scores of the participants. A one‐unit increase in the eHEALS scores of the participants corresponded to a 0.228‐unit increase in their AStSdP scores, a one‐unit increase in age corresponded to a 0.187‐unit increase in their AStSdP scores, and a one‐unit increase in their monthly income corresponded to a 0.183‐unit increase in their AStSdP scores (Table 4).

## 4 Discussion

In this study, it was determined that the eHealth literacy levels of pregnant women were “high,” and their attitudes toward sexuality were “positive.” As the eHealth literacy levels of the participants increased, their positive attitudes toward sexuality during pregnancy also increased. eHealth literacy was identified as a significant positive predictor of the attitudes of pregnant women toward sexuality. Similarly, in other studies conducted in Turkey, the eHealth literacy levels of pregnant women have been identified to be “high” (Avçin and Can 2023; Baltacı et al. 2023; Şahin et al. 2023; Yılar Erkek and Öztürk Altınayak 2024). In a study carried out in the United Arab Emirates, it was found that 71.6% of pregnant women had sufficient levels of health literacy (Elbarazi et al. 2024). In a study in Iran, it was reported that pregnant women had “good” levels of eHealth literacy (Rahdar et al. 2023). The result of this study may be interpreted from two points of view. First, the fact that the women in the sample of this study were mostly young (41.8%: 17–25 years old, 45.8%: 26–35 years old) and had high levels of education (37.0%: high school, 20%: university or higher) may have resulted in their high levels of eHealth literacy. Other studies in the literature have also shown that women at younger ages and with higher education levels search information on the internet more frequently (Taştekin Ouyaba and İnfal Kesim 2021), and their eHealth literacy is high (Şahin et al. 2023). Second, it was reported that the COVID‐19 pandemic raised the eHealth literacy of individuals by limiting their social activities and interpersonal relationships (Liang et al. 2021; Liu et al. 2021). Considering the dates on which the data of this study were collected, the eHealth literacy of the participants may have increased as a consequence of the COVID‐19 pandemic and has remained high since then.

The attitudes of the participants of this study toward sexuality during their pregnancy were determined as “positive.” The attitudes of pregnant women toward sexuality in Turkey have been reported to be “positive” in some studies (Alan Dikmen et al. 2023; Akın and Çelik 2024; Altınayak and Özkan 2024) and “negative” in some others (Güney and Bal 2023; Yılmaz Sezer et al. 2024; Yuvarlan and Beydağ 2024). Studies carried out in different cultures have, similarly, also demonstrated positive attitudes among pregnant women toward sexuality during pregnancy (Adegboyega 2019; Igbana et al. 2021; Fatima et al. 2022). In a qualitative study, while some pregnant women stated that physical changes affected their sexuality negatively, others said their sex drive increased (Leite et al. 2020). In another qualitative study, Ryan et al. (2022) reported that some pregnant women thought sexual activity during pregnancy could harm the baby and would be a sin. Other pregnant women in the same study had positive attitudes toward sexuality during pregnancy, thinking that sexual activity could help the labor process, sexual intercourse could be good for fetal health, and it is necessary for the continuation of their relationships with their partners. It is believed that the result we obtained in this study was associated with the adjustment of the participants to the changes occurring in their sexuality during pregnancy. On the other hand, it was stated in the literature that married couples could avoid sex during pregnancy because of their negative attitudes toward the physical and psychological changes occurring during pregnancy (Erbil 2019; Gümüşay et al. 2021).

It was found in this study that as the eHealth literacy levels of the participants increased, their positive attitudes toward sexuality during pregnancy also increased. Additionally, the eHealth literacy variable was identified as a factor that significantly influenced the attitudes of the participants toward sexuality. Barikani et al. (2023) reported a weak positive relationship between the health literacy levels and sexual functions of women. Dehghankar et al. (2022) revealed sexual health literacy as one of the factors that affected the sexual functions of women. In the same study, it was shown that having excellent, adequate, and somewhat inadequate sexual health literacy levels affected sexual function 4.222, 2.219, and 1.313 times more, respectively, than having very inadequate literacy levels (Dehghankar et al. 2022). In another study, Panahi et al. (2021) listed sexual health literacy among the factors that affected the sexual quality of life of women. In their study, they reported 3.415, 2.304, and 1.412 times higher sexual quality of life, respectively, in those with excellent, adequate, and somewhat inadequate sexual health literacy compared to those with very inadequate literacy levels (Panahi et al. 2021). The results of this study were in agreement with those in the literature.

This study is the first study to investigate the relationship between the eHealth literacy of pregnant women and their attitudes toward sexuality during pregnancy. For this reason, the strongest aspect of this study is its contribution to filling this gap in the literature. Nevertheless, the absence of another study on this particular topic may limit the comparability of the results and highlight the need for more studies in this field. Another important limitation of the study was that it was conducted at a single center. This limits the generalizability of its results. Finally, the data of the study were collected based on self‐reports. Because the Cronbach's alpha coefficients of the scales used to collect data were high (eHEALS: 0.951, AStSdP: 0.90), it was assumed that this limitation had been controlled for.

## 5 Conclusion

In this study, it was determined that the eHealth literacy levels of pregnant women were “high,” and their attitudes toward sexuality were “positive.” The eHealth literacy levels of the participants were identified as a significant and positive predictive factor of their attitudes toward sexuality during pregnancy. The constant advancements in technology and continuously increasing internet usage rates result in the higher abundance and complexity of information. To have the ability to protect and improve maternal and fetal health by making appropriate interventions, it is important for health professionals to know the information requirements and sources of pregnant women. eHealth literacy is an important factor for pregnant women to access information about sexuality, research such information, and make advisable decisions. Thus, healthcare professionals should evaluate the eHealth literacy levels of pregnant women and their attitudes toward sexuality during their prenatal follow‐up visits.

## Author Contributions

**Elif Çini**: conceptualization, investigation, funding acquisition, writing–original draft, methodology, validation, visualization, writing–review and editing, software, formal analysis, project administration, data curation, supervision; resources. **Hamide Aygör**: conceptualization, investigation, writing‐original draft, methodology, validation, visualization, writing–review and editing, software, formal analysis, project administration, data curation, supervision, resources.

## Ethics Statement

The study was approved by Necmettin Erbakan University Health Sciences Scientific Research Ethics Committee (Approval no. 221), and necessary permissions were obtained from the hospital where the study was carried out.

## Conflicts of Interest

The authors declare no conflicts of interest.

### Peer Review

The peer review history for this article is available at https://publons.com/publon/10.1002/brb3.70390.

## Supporting information



Supporting Information

## Data Availability

The data that support the findings of this study are available from the corresponding author upon reasonable request.
