# Supplementary material for: Relationship Between Pregnant Women's eHealth Literacy and Their Attitudes Toward Sexuality
Source: Brain Behav. 2025 Feb 28;15(3):e70390. doi: 10.1002/brb3.70390 (PMC11870822; doi:10.1002/brb3.70390)
Supplement: Supplementary file 1 — Supporting Information [file BRB3-15-e70390-s001.pdf]

GLOCAL TRANSLATION
THINK GLOBAL ACT GLOCAL

22.10.2024

Declaration:

The article titled **"The relationship between pregnant women's eHealth literacy and their attitudes towards sexuality"** was edited/proofread by a native speaker. To the best of the Editor's knowledge, the article does not contain any mistakes in terms of language. Editing was restricted to language and other structural criteria determined/requested/required by the author/publication, and it did not interfere with any semantic or factual aspects, statements, or findings of the research reported in the text.

Proofreader:

Andrew Harmon

Glocal Translation

İstanbul

Glocal & INDEX
Emaar Residence İç Kapı No:291 Üsküdar/İST.
Vergi No: 9130292628 Tel: (216) 232 26 02
www.indextercume.com

GLOCAL TRANSLATION
THINK GLOBAL ACT GLOCAL
